# Anti-Bacterial Activity of Green Synthesised Silver and Zinc Oxide Nanoparticles against *Propionibacterium acnes*

**DOI:** 10.3390/ph17020255

**Published:** 2024-02-16

**Authors:** Hafez Al-Momani, Muhannad I. Massadeh, Muna Almasri, Dua’a Al Balawi, Iman Aolymat, Saja Hamed, Borhan Aldeen Albiss, Lugain Ibrahim, Hadeel Al Balawi, Sameer Al Haj Mahmoud

**Affiliations:** 1 Department of Microbiology, Pathology and Forensic Medicine, Faculty of Medicine, The Hashemite University, Zarqa 13133, Jordan; 2 Department of Biology and Biotechnology, Faculty of Science, The Hashemite University, Zarqa 13115, Jordan; massadeh@hu.edu.jo (M.I.M.); monaalmasri23@yahoo.com (M.A.); 3 Faculty of Applied Medical Sciences, The Hashemite University, Zarqa 13133, Jordan; 4 Department of Anatomy, Physiology and Biochemistry, Faculty of Medicine, The Hashemite University, Zarqa 13133, Jordan; imank@hu.edu.jo; 5 Department of Pharmaceutical & Pharmaceutical Technology, Faculty of Pharmaceutical Sciences, The Hashemite University, Zarqa 13133, Jordan; hamedsh@hu.edu.jo; 6 Nanotechnology Institute, Jordan University of Science & Technology, Irbid 22110, Jordan; baalbiss@just.edu.jo; 7 Department of Basic Medical Science, Faculty of Medicine, Al-Balqa’ Applied University, AL-Salt 19117, Jordan; sameer.alhaj@bau.edu.jo

**Keywords:** acne vulgaris, biofilm, *Propionibacterium acne*, silver, zinc oxide, nanoparticles

## Abstract

*Propionibacterium acnes* plays a critical role in the development of acne vulgaris. There has been a rise in the number of patients carrying *P. acnes* strains that are resistant to antibiotics. Thus, alternative anti-microbial agents are required. Zinc oxide (ZnO-NPs) and silver (Ag-NPs) nanoparticles can be used against several antibiotic-resistant bacteria. The impact of Ag-NPs and ZnO-NPs against two clinical strains of *P. acnes*, P1 and P2, and a reference strain, NCTC747, were investigated in this research. A chemical approach for the green synthesis of Ag-NPs and ZnO-NPs from *Peganum harmala* was employed. The microtiter plate method was used to examine the effects of NPs on bacterial growth, biofilm development, and biofilm eradication. A broth microdilution process was performed in order to determine minimal inhibitory (MIC) concentrations. Ag-NPs and ZnO-NPs had a spherical shape and average dimensions of 10 and 50 nm, respectively. MIC values for all *P. acnes* strains for Ag-NPs and ZnO-NPs were 125 µg/mL and 250 µg/mL, respectively. Ag-NP and ZnO-NP concentrations of 3.9- 62.5 µg/mL and 15–62.5 µg/mL significantly inhibited the growth and biofilm formation of all *P. acnes* strains, respectively. ZnO-NP concentrations of 15–62.5 μg/mL significantly inhibited the growth of NCTC747 and P2 strains. The growth of P1 was impacted by concentrations of 31.25 μg/mL and 62.5 μg/mL. Biofilm formation in the NCTC747 strain was diminished by a ZnO-NP concentration of 15 μg/mL. The clinical strains of *P. acnes* were only affected by ZnO-NP titres of more than 31.25 μg/mL. Established *P. acne* biofilm biomass was significantly reduced in all strains at a Ag-NP and ZnO-NP concentration of 62.5 µg/mL. The findings demonstrated that Ag-NPs and ZnO-NPs exert an anti-bacterial effect against *P. acnes*. Further research is required to determine their potential utility as a treatment option for acne.

## 1. Introduction

The human dermis is a particularly vulnerable structure which may allow a range of bacteria to gain entry into the body [1]. Acne vulgaris is one of the most frequently arising skin pathologies in young adults aged between 11 and 30 years, and typically affects 50.9% of females and 42.5% of males [2,3]. The presentation of acne can be affected by numerous factors, including hormone titres and variations in keratinisation, as well as altered inflammatory or immune activity [4,5]. It has been recognised that the degree of inflammation may be influenced by the reaction of the host to the bacterium, *P. acnes*, formerly known as *Corynebacterium acne* [6], which liberates free fatty acids as a result of sebaceous triglyceride metabolism [7]. The latter cause irritation of the follicular wall and neighbouring dermal layer. *P. acnes* also produces exoenzymes and chemoattractants which target neutrophils [5,7]. It has been demonstrated that diminishing the bacterial population of *P. acnes* on the skin can be a successful treatment [8].

Antibiotics are frequently used topically and orally to treat acne [9]. According to data from multiple studies, the percentage of patients who have *P. acnes* strains that are resistant to one or more medications has dramatically increased in recent years [10,11,12,13,14]. This may be due to the fact that cells of *P. acnes* present within the pilosebaceous unit develop and form a biofilm [15]. Genome sequencing of *P. acnes* shows three distinct clusters of genes that encode enzymes involved in the extracellular polysaccharide production necessary for biofilm formation [6]. The most significant property of biofilm, and the main contributor to persistent infections, is its resistance to anti-microbial drugs and to elements of the human immune system [16,17,18]. In the treatment of acne, the use of nanoparticles (NPs) is a promising method with which to overcome bacterial resistance [19,20].

NPs exhibit considerable potential for use as anti-bacterial treatments, offering a broad spectrum of activity and appearing resilient to the development of bacterial resistance to their anti-microbial effects. These therapeutic properties arise as a result of their small size and consequent high surface/volume ratio, which enable NPs to interact with bacterial membranes [21]. Both organic and inorganic NPs exist; examples include liposomes or metallic NPs, respectively. These two types of NPs have been successfully utilised for a number of clinical disorders, with metallic NPs demonstrating the greatest therapeutic potential [22]. The latter have a range of effects on a number of bacteria which exhibit resistance to multiple conventional anti-bacterial agents [23]. There is therefore considerable interest in exploiting metallic NPs in both biomedical and pharmaceutical domains [24,25].

The most widely studied metal NPs are silver (Ag) NPs (Ag-NPs). Although Ag ions and substances based on Ag exhibit extreme toxicity to a number of bacteria, the metal’s anti-bacterial properties are markedly improved when Ag-NPs are utilised, owing to the resulting surface area augmentation [26,27,28]. Anti-microbial properties have also been demonstrated in metallic oxide NPs, e.g., zinc oxide NPs (ZnO-NPs) [22]. The latter have been investigated in detail owing to their specific physiochemical characteristics, as they exhibit effects which counter bacterial infection, malignancy, and inflammation [29,30]. Earlier studies have shown that when used at specific doses, ZnO-NPs may be of value for use as clinical anti-bacterial agents [31,32].

The biomedical and anti-bacterial characteristics of NPs are, in general, a consequence of the techniques and chemicals used for their manufacture and formulation, e.g., temperature, concentration, and the reducing agent and solvent utilised [33,34]. A green synthesis strategy has been applied to generate NPs that exhibit biocompatibility and biodegradability which make them suitable for various biomedical applications [35]. This environmentally friendly, sustainable, and dependable method of manufacture also circumvents the creation of undesirable or noxious by-products [36,37,38]. Plant extracts have been studied in depth for use in this process owing to their ability to enhance NP monodispersity. They contain a range of biomolecules, such as phenolics, polysaccharides, flavones, terpenoids, alkaloids, proteins, amino acids, enzymes, and alcoholic compounds, which can function as reducing and capping agents, the latter contributing to the stability and controlling the configuration of the produced NPs [39,40].

A member of the Zygophyllaceae family, harmala (*P. harmala)*, is recognised for its medicinal properties. Its 5 mm diameter seeds are black in colour, globular in shape, and have a spicy taste and nut-like scent. They are frequently used as a condiment in cooking. Clinically, they have been utilised therapeutically for conditions including back pain, bronchospasm, colic, and jaundice, and to promote menstruation [41,42]. The activity of *P. harmala* against disease-inducing pathogens, such as bacteria, fungi, and viruses, has also been documented [42,43]. It has been hypothesised in this research paper that the pharmacological properties of Ag-NPs and ZnO-NPs synthesised using *P. harmala* seed extract as a reducing agent could be used to generate potent cosmeceutical products that could be utilised as acne treatments. The effects of Ag-NPs and ZnO-NPs on the growth and biofilm formation of *P. acnes* have therefore been examined in this study.

## 2. Methods

### 2.1. Propionibacterium Acne Strains and Culture Conditions

Two clinical strains of *P. acnes* were acquired from the Prince Hamza Hospital and identified by their analytical profile index (BioMerieux, Marcy-l’Etoile, France). A reference strain, NCTC747, was also used in this study. *P. acnes* strains were cultured in tryptic soy broth (TSB) at 37 °C under anaerobic conditions using an OXOID anaerobic jar and AnaeroGen atmosphere generation systems. Written informed consent was obtained from the patients whose clinical isolates were obtained, and all ethical guidelines were properly observed. The ethics service committees from both Hashemite University and the Prince Hamza Hospital granted ethical approval for this case study (reference number 5/3/2020/2021).

### 2.2. Green Synthesis of Silver and Zinc Oxide Nanoparticles

#### 2.2.1. Preparation of Aqueous *P. Harmala* Seed Extract

The first researcher obtained whole *P. harmala* plants from their own farm in the Abbin region of Ajloun Governorate in September 2023. The identification of the *P. harmala* seeds was verified by comparing them to the sample at the herbarium of Jerash University’s Faculty of Agriculture and confirmed by the Assistant Professor of Agriculture. A voucher specimen (AM/2023/01/002) was then stored at the herbarium within the Plant Protection Department at Jerash University in Jordan. The harvesting of *P. harmala* followed institutional, national, and international guidelines and laws.

After being fully dried at room temperature and cleaned with double-distilled water (Sigma-Aldrich, Darmstadt, Germany), the *P. harmala* seeds were machine-ground into a coarse powder. A total of 5 g of powder was combined with 50 mL of double-distilled water and heated to 75 °C for 90 min before being filtered twice through Whatman filter paper. Using microfiltration through a 0.22 mm syringe membrane filter, a clear aqueous extract with a pH of 6.4 at 25 °C was obtained.

#### 2.2.2. Determination of Total Phenolic and Total Flavonoid Content in the *P. harmala* Extract

The Folin–Ciocalteu technique [44] was utilised in order to quantify the phenolic compounds, i.e., secondary metabolites from plants characterised by their ability to scavenge radicals as result of their redox potentials [45], in the extract of *P. harmala.* This investigation involves phenolic moiety oxidation following the addition of Folin–Ciocalteu reagent, which comprises phosphomolybdic and phosphotungstic acids [46]. Following the published method by Singleton, Orthofer, et al. [44] with some modifications, 100 μL Folin–Ciocalteu reagent was combined with 20 μL *P. harmala* extract. A total of 300 μL 25% sodium carbonate solution was admixed after 10 min. The absorbance of the solution was then determined at a wavelength of 765 nm. This experiment was performed in triplicate and the mean absorbance value was calculated. Solutions of gallic acid of strengths 0–500 mg/L were utilised to determine the calibration curve. Data are expressed as gallic acid equivalents (GAE) per 100 gm extract.

The number of flavonoids within the sample were also established using spectrophotometry. The technique used relied on the production of a flavonoid–aluminium complex [47], which demonstrated maximum absorption at a wavelength of 430 nm. A calibration was constructed using quercetin. A total of 1 mL of both extracts of *P. harmala* and 2% aluminium chloride methanolic solution were admixed, and then incubated for 10 min in ambient conditions. The solution’s absorbance was quantified at the above wavelength. Data are expressed as quercetin equivalents (QE) per 100 gm extract.

#### 2.2.3. Green Synthesis of Silver and Zinc Oxide Nanoparticles

Ag-NPs were produced by heating a 50 mL solution of 3 mM AgNO_3_ to 80 °C in a 100 mL foil-lined Erlenmeyer flask and stirring it constantly at 1100 rpm. A 4 mL aqueous *P. harmala* seed extract solution was applied using a micropipette at a rate of 34 µL/min. The colour of the solution changed from orange to dark brown immediately as the Ag-NPs started to form. Ag-NPs are created through the reduction of silver ions, and this process was observed using ultraviolet (UV) spectrophotometry, which measured the absorbance of the reaction medium in the 300–800 nm wavelength range. Centrifugation was performed for 15 min at 10,000 rpm to purify the synthesised Ag-NPs. The supernatant was placed in a dry, clean container to enable particles to settle further, after which centrifugation was performed repeatedly using a cooling microfuge. This enabled the Ag-NPs to be dried, purified, and characterised.

In order to produce ZnO-NPs, 50 mL aqueous zinc sulphate heptahydrate solution (ZnSO_4_·7H_2_O, ACS reagent, 99%, Sigma-Aldrich, St. Louis, MO, USA) was combined with 5 mL aqueous yellow seed extract solution from *P. harmala*. The mixture was then agitated at room temperature for 10 min, creating a pale-yellow solution. After a thorough washing process, the obtained NP solutions were centrifuged at 4500 rpm for 15 min and then dried at 80 °C for 7–8 h. Before usage, the crude pellets were resuspended in sterile double-distilled water, filtered through a 0.2 µm filter, and stored in the dark at a temperature of 4 °C. Once they had been dispersed in sterile, double-distilled water and centrifuged three times, the suspended particles were filtered. This resulted in the formation of ZnO-NPs.

### 2.3. Characterisation of Zinc Oxide and Silver Nanoparticles

#### 2.3.1. X-ray Diffraction

An X-ray diffractometer (XRD-6000, Shimadzu, Kyoto, Japan)), equipped with a copper K-α radiation source with a wavelength of 0.154 nm, was employed to measure the X-ray diffraction (XRD) spectra of the nanoparticles. The sample holder was 2 cm long and 0.5 mm wide.

#### 2.3.2. Analysis of Particle Sizes

A Malvern Zetasizer Nano ZS90 (Malvern Ltd., Malvern, UK) was used to measure particle sizes. The stock suspensions were diluted in distilled water. In order to guarantee temperature homogeneity, particle samples were allowed to equilibrate at 25 °C for 5 min before duplicate tests, including three separate trials, were performed. The viscosity and refractive index values of the solution used were 0.8872 cP and 1.59, respectively. The tests and experiments were performed at the Nanotechnology Institute at Jordan University of Science and Technology.

#### 2.3.3. Ultraviolet–Visible Spectroscopy

UV–visible (UV-Vis) spectroscopy (UV-1900, Shimadzu, Kyoto, Japan) was used to track the bioreduction of the AgNO_3_ salt solution and to examine the production of the Ag-NPs and ZnO-NPs using *P. harmala* seed aqueous extract. Double-distilled water was employed as a control. All spectrophotometric analyses were carried out inside a quartz cuvette with a path length of 1 cm. The surface plasmon resonance (SPR) of the NPs was measured using a wavelength range of 800–200 nm.

#### 2.3.4. Scanning Electron Microscopy

In order to examine the surface size and characteristics of the biosynthesised NPs, a scanning electron microscope (SEM) was employed. A fine 4 nm layer of gold was applied to the surface of the samples, after which they were evaluated using a low vacuum of 50 Pa and 3 KV, which had a working distance of 8–10 mm.

### 2.4. Anti-Acne Effect of Synthesised Silver and Zinc Oxide Nanoparticles

#### 2.4.1. Agar Well Diffusion Approach

In order to determine the efficacy of the Ag-NPs and ZnO-NPs against *P. acnes,* the agar well diffusion technique was applied. During this experiment, a single colony from each strain was incubated under ideal anaerobic conditions for 72 h at 37 °C. This led to the generation of a standardised culture, 1.5 × 10^8^ CFU/mL, of each bacterium. Following incubation, a spectrophotometer, measuring optical density (OD) at a wavelength of 600 nm, was used to adjust the culture turbidity to 0.5 MFU. Sterile cotton swabs were used to distribute 100 μL of the previously generated standardised culture for each bacterium onto the Mueller–Hinton agar plates. Wells of 6 mm were punched into the agar plates containing the bacterial lawn, into which 40 μL of each 250 µg/mL NP solution was poured. The plates were kept for 10 min at room temperature so as to allow the NPs to diffuse into the agar bacterial lawn. The positive and negative controls were double-distilled, and then antibiotic discs containing doxycycline were added. Following incubation of each plate in the previously recommended ideal conditions, a growth inhibition zone was determined and measured. Each experiment was performed twice.

#### 2.4.2. Determination of Minimum Inhibitory and Bactericidal Concentrations Using the Broth Microdilution Method

The minimum bactericidal concentration (MBC), i.e., the lowest concentration of anti-microbial agent needed to kill microorganisms, and the minimum inhibitory concentration (MIC), i.e., the lowest concentration of anti-microbial agent required to prevent microbial growth, were determined in this experiment. According to Clinical and Laboratory Standards Institute procedure, under ideal conditions, a single colony of the *P. acnes* bacterial strains should be used to create a standardised *P. acnes* culture.

Culture turbidity was adjusted to the 0.5 MFU standard by applying a spectrophotometer to measure the absorbance. This was then diluted with sterile Mueller–Hinton Broth (MHB) so as to create a standard inoculum strength (10^5^ CFU/mL) over a 15 min period. Subsequently, 180 µL of sterile MHB was added to 96-well plates; 200 µL of Ag-NPs and ZnO-NPs stock solutions (1000 µg/mL) were placed into the first well. Subsequently, with each progressive well up until the ninth well, two-fold serial dilutions were performed with up and down mixing in order to produce NP solution concentrations of 1.9, 3.9, 7.8, 15.625, 31.25, 62.5, 125, 250, and 500 µg/mL.

A total of 20 µL from earlier bacteria standard cultures was added to each of the wells with the exception of wells 10 and 12, which were kept in place in order to maintain media sterility. The 96-well *P. acnes* plates underwent anaerobic incubation for 72 h at 37 °C. The lowest NP concentration, where there was no visible growth in the wells, was the MIC endpoint. The MBC endpoint was considered to have been reached when 99.9% of the bacteria had been killed in the presence of the lowest NP concentration. This was achieved by evaluating the volume of bacteria present on agar plates before and after incubation. The experiments were repeated twice.

#### 2.4.3. Bacterial Growth Assays

Various titres of Ag-NPs and ZnO-NPs were studied to assess their impact on bacterial growth and biofilm development using 96-well, flat-bottomed, transparent, and sterile microtiter plates (BD Falcon Franklin Lakes, NJ, USA). For the growth assay, 180 µL aliquots of TSB were used to prepare standardised suspensions of *P. acnes* cultures; the latter were added to 20 µL bacterial aliquots suspended in TSB and 200 µL of either Ag-NPs or ZnO-NPs, i.e., 200 µL of 125 g/mL in the first well, which was then double-diluted to concentrations ranging from 0.79 µg/mL to 62.5 µg/mL. Each test was carried out three times.

Control wells were also employed, with positives containing *P. acnes* and TSB, and negatives containing only TSB. The samples were then subjected to anaerobic incubation in the dark at 37 °C and shaken for 72 h. After the incubation process was completed, the total cell numbers at different NP concentrations were determined by measuring the optic density (OD) at 600 nm (OD600, Infinite^®^ 200 PRO NanoQuant, TECAN, Austria). In order to calculate the final cell growth value, the average reading from each well containing *P. acnes* was subtracted from the average reading of the wells without *P. acnes* (blanks) at the relevant Ag-NP concentration. The total cell growth measured was that for all cells, i.e., biofilm-associated and planktonic. At the end of the incubation period, 100 μL of the culture was spread over a nutrient agar plate and incubated at 37 °C for 72 h under anaerobic conditions using an OXOID anaerobic jar.

#### 2.4.4. Quantitative Determination of Biofilm Formation via Microtiter Plate Assessments

A spectrophotometric method was used to compute the total biofilm biomass, counting bacterial cells and extracellular polymeric substances (EPSs) [48]. Similar to the procedure used for the growth assay, after a 72 h incubation period without shaking, the wells were then shaken and washed with 250 µL sterile phosphate buffer saline in order to eliminate any bacteria that had not adhered to the wells.

A total of 200 µL 99% methanol was used to fix the microorganisms in each well. Before being emptied and given time to dry, the initial plates were allowed to stand for 15 min, following which they were stained for 5 min using 200 µL 2% Hucker crystal violet, a stain that is suitable for Gram-staining the contents of each individual well. The wells were cleaned three times in 200 µL of sterile water to eliminate any remaining staining material. Care was taken not to disturb the biofilm with each wash. Following another period of drying, the cell-bound dye on the plates was redissolved in 33% (*v*/*v*) glacial acetic acid at a rate of 160 µL per well.

An automated reader (ICN Flow Titertek Multiscan Plus McLean, VA, USA)) was used to determine the OD of each well. The measurements were taken three times, i.e., before the samples were incubated (OD, 600 nm), during sample incubation at the growth evaluation stage (OD, 600 nm), and after the biofilm assay had been created (OD, 570 nm). A ratio of 570:600 was selected in order to normalise the quantity of biofilm that had developed in comparison to the rate of bacterial development. An OD value that was negative was shown as 0. Each test was carried out three times. A cut-off value (ODc) was confirmed, which was three standard deviations (SDs) higher than the mean OD of the negative control, i.e., ODc  =  average OD of negative control  +  3SDs of negative control. Based on the OD, the isolates were assigned to one of four categories, i.e., non-biofilm producer (OD  <  ODc), weak biofilm producer (ODc  <  OD  <  2ODc), moderate biofilm producer (2ODc  <  OD  <  4ODc), and strong biofilm producer (4ODc  <  OD).

#### 2.4.5. Microtiter Biofilm Eradication Test

A process combining crystal violet (CV) staining with a tetrazolium dye was used to evaluate the effects of both Ag-NPs and ZnO-NPs on already formed biofilms. As cellular nicotinamide adenine dinucleotide and hydrogen drives the creation of colourful formazan salts, tetrazolium salts are frequently used as reagents in biological tests to assess the metabolic behaviour of living cells [49]. Various tetrazolium-based dyes, therefore, have been utilised to identify and to evaluate biofilms [50]. In this work, triphenyl tetrazolium chloride (TTC) was employed as a metabolic marker of bacterial survival and biofilm development.

In order to create biofilms, standardised bacterial suspensions were prepared. Seventy-two-hour cultures of each *P. acnes* strain were carried out on 2 different 96-well plates, using TSB at a concentration of 0.5 MFU. Aliquots of 20 µL of the bacterial suspension were introduced to 250 µL aliquots of TSB, containing no Ag-NPs, to produce biofilms. Ultimately, this produced a 270 µL volume in the microtiter well. The plates were incubated anaerobically for 72 h at 37 °C to promote biofilm attachment and growth. Two wells were placed on separate, parallel microtiter plates for each concentration that was tested; one was stained with CV, and TTC was added to the other.

The media were taken off the plates over the next three days, after which the plates were placed upside down on sterile paper and left to dry for 15 min at room temperature. Subsequently, a multi-channel pipette was used to remove the planktonic and unattached cells from each well, and the remaining biofilm was rinsed three times in 150 µL new, sterile media. Any excess rinsing material was aspirated out of each well before 200 µL of fresh MHB containing NP concentrations ranging from 0.97 µg/mLto 62.5 µg/mL was added. The plates were then incubated at 37 °C for 72 h. Following incubation, the planktonic cells and media were removed, the planktonic growth was estimated, and the remaining biomass was rinsed three times in distilled water.

An enzyme-linked immunosorbent assay microplate reader was then used to obtain the OD600 values of each plate, which were documented before and after incubation. The combined results from the three different biological replicate results were averaged out.

As mentioned previously, the second plate was stained with TCC. Microplates were incubated at 37 °C for 6 h after adding 40 µL 0.2 mg/mL TCC, dissolved in deionised water, to the wells of the *P. acnes*, which had been treated with various Ag-NP concentrations for 72 h. Results from a minimum of three replicates were averaged. Once treatment had been performed, the mean OD490 values for each biofilm were measured [51].

### 2.5. Statistical Analysis

The experimental findings were presented as the mean standard error of the mean of at least three replicates. One-way analysis of variance (ANOVA) was used to assess differences between samples and controls where pertinent. The OD values from the microtiter plate tests with and without Ag-NP treatment at different dilutions were compared using the Mann–Whitney U test or Kruskal–Wallis test followed by post hoc Dunn’s multiple comparisons. Significant *p* values were determined to be less than 0.05. GraphPad Instat 6.0 software was used to analyse the data.

## 3. Results

### 3.1. Determination of Total Phenolic and Flavonoid Content

The Folin–Ciocalteu method determined that the samples had a total phenolic content of 8.11 ± 1.7 mg GAE/100 mg extract. Spectrophotometric analysis following the colorimetric aluminium chloride assay revealed a total flavonoid content in the samples of 0.76 ± 0.12 mg QE/100 mg extract.

### 3.2. Nanoparticle Characterisation

#### 3.2.1. Ultraviolet–Visible Spectroscopy

During their preparation, the formation of Ag-NPs causes a colour change in the reaction mixture from orange to dark brown, indicating AgNO_3_ reduction in the Ag-NPs. During the preparation of the ZnO-NPs, the solution mixture of ZnSO_3_ and *P. harmala* seed extract changed from light brown to yellowish-black in colour, indicative of the formation of ZnO-NPs (Supplementary Figure S1).

**Figure 1:**
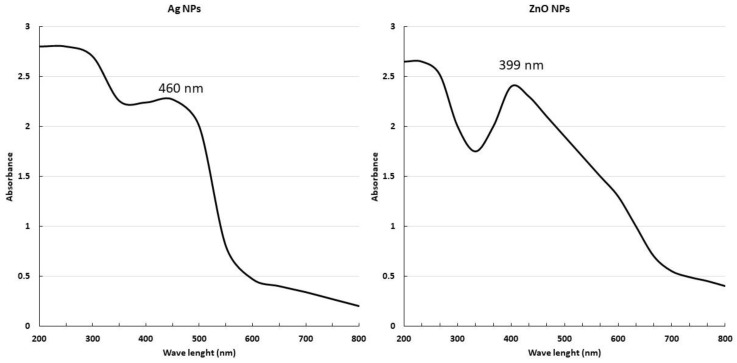
Characterisation of NPs by ultraviolet–visible spectroscopy.

The green synthesised Ag-NPs and ZnO-NPs were characterised using a UV-Vis spectrophotometer in the wavelength range 800–200 nm. As both Ag-NPs and ZnO-NPs are direct bandgap semiconductors, with a wide bandgap of ~3.37 eV between the conduction and the valence bands [52], the electron jump from the valence band to the conduction band generates an absorption peak between 450 and 500 nm, and between 350 and 450 nm, respectively. The UV-Vis spectrum shows a large absorption peak at approximately 460 nm for Ag-NPs, and at 399 nm for ZnO-NPs (Figure 1).

#### 3.2.2. Scanning Electron Microscopy

**Figure 2:**
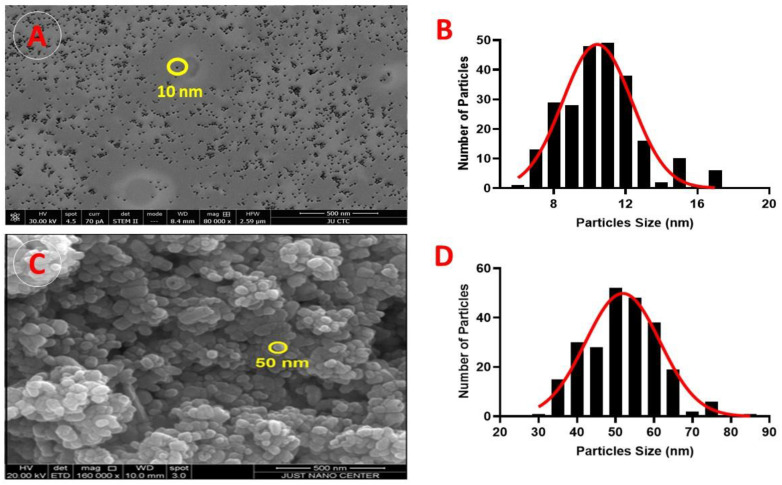
Scanning electron microscopy (SEM) micrographs: (**A**) SEM image (×500 magnification) of Ag-NP morphology; yellow circles indicate the circumference of the NPs. (**B**) SEM results of particle size distribution of Ag-NPs. (**C**) SEM image (×500 magnification) of ZnO-NP morphology; yellow circles indicate the circumference of the NPs. (**D**) SEM results of particle size distribution of ZnO-NPs.

The size and surface morphology of the nanoparticles were assessed using SEM. Figure 2A reveals the morphological characteristics of the Ag-NPs; they have an average particle size of approximately 10 nm, are of a uniform spherical shape, and do not aggregate. The size histograms of the Ag-NPs are displayed in Figure 2B and show that the main particle sizes for the Ag-NPs produced in this experiment were 10.9 ± 2.7 nm.

The SEM images of the ZnO-NPs demonstrated that these NPs were uniformly shaped and sized, measuring, on average, 50 nm (Figure 2C). Figure 2D displays the size histograms for the ZnO-NPs; these have a main particle size of 49.8 ± 12.6 nm.

#### 3.2.3. X-ray Diffraction

The samples were left at an ambient temperature of 24.85 °C in order to investigate the formation of crystalline phases in the synthesised Ag-NPs and ZnO-NPs using copper K-α radiation, with a wavelength of 1.5406 Å in the (2θ) range from 10 to 80.

**Figure 3:**
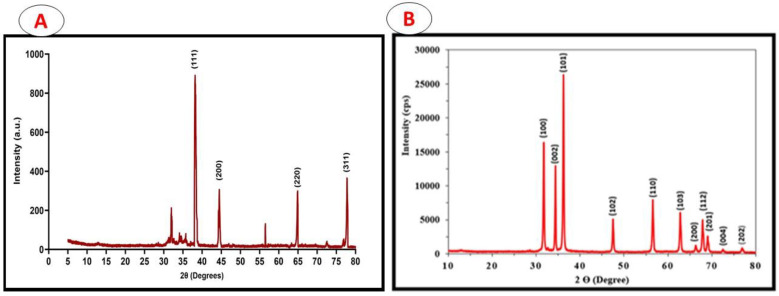
X-ray diffraction showing crystalline phases of the synthesised Ag-NPs (**A**) and ZnO-NPs (**B**).

The Ag-NPs showed four peaks at the 2θ values, 38.2901, 44.5583, 64.8185, and 77.4383 (Figure 3A). These were considered to represent the Ag metal and the corresponding Ag plane values of hkl, which are (111), (200), (220), and (311), respectively. The diffractogram (Figure 1) and the Joint Committee on Powder Diffraction Standards (JCPDS), silver file No. 04-0783, were compared and demonstrated the face-centred cubic configuration of the Ag-NPs. The Scherrer equation [53] was applied to compute the Ag-NPs’ particle dimension, which was determined to be in the region of 13.9 nm.

In the ZnO-NP sample, the Miller indices h, k, and l, i.e., (100), (002), (101), (102), (110), (103), (200), (112), (201), (004), and (202) [JCPDS No. 36-1451], were associated with the typical hexagonal wurtzite ZnO-NP architecture reflecting the orientation of the crystals. The dimensions of the latter were shown to be 44 nm by entering the dominant peaks of diffraction, essentially 101, into the Debye–Scherrers equation. These results confirm that the Ag-NPs and ZnO-NPs produced were of a high quality, containing no contaminants within the crystalline configuration.

#### 3.2.4. Particle Size and Zeta Potential Analysis

**Figure 4:**
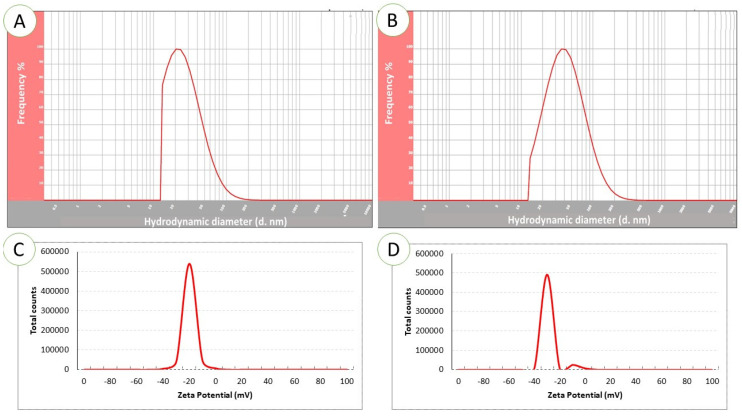
Dynamic light scattering (DLS) spectrum showing the particle size distribution of Ag-NPs (**A**) and ZnO-NPs (**B**). Zeta potential analysis of Ag-NPs (**C**) and ZnO-NPs (**D**).

Dynamic light scattering demonstrated mean Ag-NP particle dimensions to be 22 nm (Figure 4A) and mean ZnO-NP particle size to be 48 nm (Figure 4B). The electrical potential which arises as a result of the relative motions of the NP and solvent at the solid–liquid interface is referred to as the zeta potential. The electrical potential and the surface charge influence NP stability. The Ag-NPs were determined to have a zeta potential of −25.3 Mv (Figure 4C); the zeta potential of the ZnO-NPs was −34.7 Mv (Figure 4D).

### 3.3. Anti-Bacterial Activity of Silver and Zinc Oxide Nanoparticles against P. acnes

#### 3.3.1. Well Diffusion Assay

Both Ag-NPs and ZnO-NPs showed encouraging anti-microbial properties when tested in the presence of all of the *P. acnes* strains under investigation (Table 1). After taking into account the inhibitory zones, the *P. acnes* reference strain, NCTC747, showed the maximum sensitivity towards Ag-NPs, with an inhibition zone of 13.2 ± 0.8 mm. P1 and P2 measurements were 11.7 ± 0.8 mm and 11.5 ± 0.5 mm, respectively. For NCTC747, P1, and P2, the mean inhibition zone measurements using ZnO-NPs were 11.0 ± 1.0, 9.7 ± 1.2 mm, and 9.7 ± 0.6 mm, respectively.

**Figure 5:**
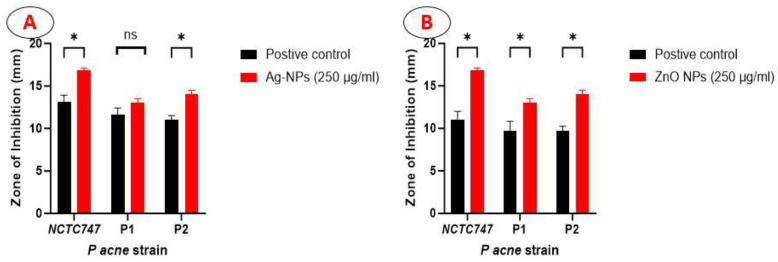
Bar graph showing the diameter of the zone of inhibition produced by Ag-NPs (**A**) and ZnO-NPs (**B**) at a concentration of 250µg/mL, compared to the positive control (30 µg doxycycline).* statistically significant with *p* < 0.01, ns ( not statistically significant).

There was a significant larger zone of inhibition created by the Ag-NPs in relation to NCTC747 (t(df) = 7.778 (4.00), *p* = 0.001) and P2 strains (t(df) = 7.348 (4.00), *p* < 0.001) (Figure 5A). The ZnO-NPs showed a larger zone of inhibition compared to the positive control: NCTC (t(df) = 7.707 (4.00), *p* < 0.001); P1 (t(df) = 4.588(4.00), *p* = 0.003); P2 (t(df) = 9.827(4.00), *p* < 0.001) (Figure 5B).

#### 3.3.2. Minimal Inhibitory and Bactericidal Concentrations for Silver and Zinc Oxide Nanoparticles

After a 72 h incubation period in anaerobic conditions at 37 °C, turbidity was evident in the test tubes containing *P. acnes* strains at doses from 3.9 µg/mL to 62.5 µg/mL of Ag-NPs, indicating bacterial growth. There was no sign of turbidity in the test tubes containing the studied strains with Ag-NP concentrations of 125 µg/mL and 250 µg/mL, demonstrating that bacterial growth had been inhibited. Two brain heart infusion agar plates were inoculated, one with the suspension from the tube containing a concentration of 125 µg/mL, and the other from the tube containing 250 µg/mL. The plates were incubated for 72 h. The Ag-NPs were considered bactericidal since there was no bacterial growth on either plate; the MIC and MBC values were both 125 µg/mL. The MIC and MBC values for the ZnO-NPs were both 250 µg/mL.

#### 3.3.3. Inhibitory Effect of Silver and Zinc Oxide Nanoparticles on Planktonic Growth and Biofilm Formation of *P. acnes*

**Figure 6:**
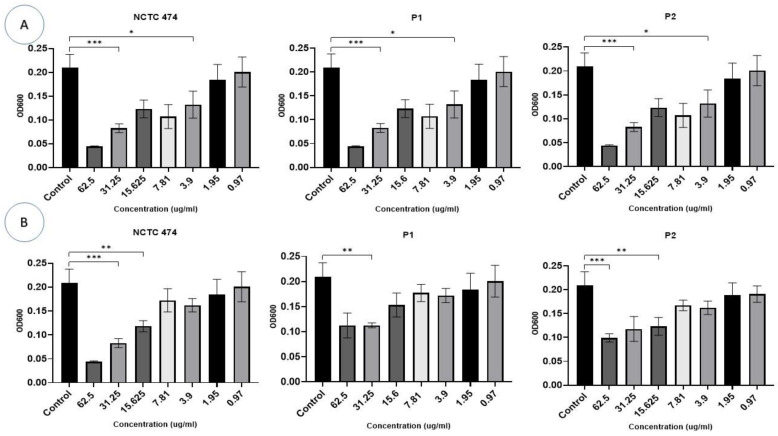
The effect of different concentrations (0.97–62.5 g/mL) of Ag-NPs (**A**) and ZnO-NPs (**B**) on the growth of *P. acne* strains after a 72 h incubation period. The OD at 600 nm is represented on the *Y*-axis. Data are calculated as mean ± standard deviation. Asterisks are used to denote the different *p* values as calculated by one-way analysis of variance (ANOVA): *** *p* <0.0001, ** *p* < 0.001, and * *p* < 0.01.

*P. acnes* strains were incubated with different concentrations (0.97–62.5 µg/mL) of Ag-NPs and ZnO-NPs for 72 h (Figure 6). Bacterial growth was identified and compared to the growth of the controls. Ag-NP concentrations >3.9 µg/mL inhibited the growth of all *P. acnes* strains after 72 h (F (10, 11) = 24.85, *p* < 0.0001) (Figure 6A).

ZnO-NP concentrations of 15–62.5 μg/mL inhibited growth in two *P. acnes* strains, i.e., NCTC747 and P2 (*p* < 0.05). The P1 strain was impacted by concentrations of 31.25 μg/mL and 62.5 μg/mL (F (10, 11) = 36.27, *p* < 0.0001). Lower Zn-NP concentrations showed a trend towards negatively affecting growth for all strains, but this failed to reach statistical significance (Figure 6B).

**Figure 7:**
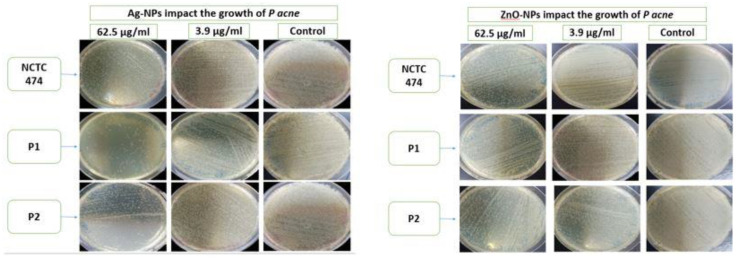
Anti-bacterial activity of Ag-NPs and ZnO-NPs at concentrations of 62.5 μg/mL and 3.9 μg/mL using the spreading method with respect to NCTC747, P1, and P2 strains.

Figure 7 illustrates the use of the spreading method to determine the anti-microbial activities of the Ag-NPs and ZnO-NPs at concentrations of 62.5 μg/mL and 3.9 μg/mL against *P. acne* in comparison to the control samples.

Based on the findings of the microtiter plate tests, it can be concluded that the NCTC747 strain was an intermediate biofilm producer, and that the remaining strains were strong biofilm producers (Supplementary Table S1).

**Figure 8:**
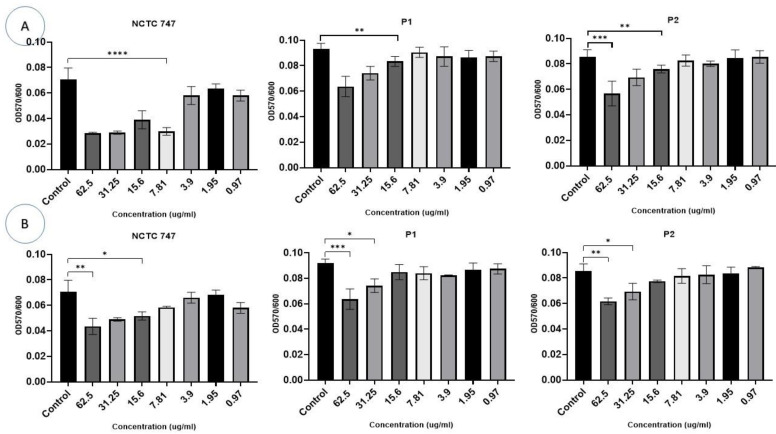
Effects of different concentrations (0.97–62.5 g/mL) of Ag-NPs (**A**) and ZnO-NPs (**B**) on biofilm development for the *P. acnes* strains following a 72 h incubation. The biomass was stained using the crystal violet process. Data are calculated as mean ± standard deviation. Asterisks are used to mark the different *p* values as calculated by one-way analysis of variance (ANOVA): **** *p* < 0.0001, *** *p* < 0.0001, ** *p* < 0.001, and * *p* < 0.01.

Biofilm formation was reduced by the incorporation of Ag-NPs at concentrations of ≥15.6 µg/mL in the NCTC747 strain (F (7, 8) = 7.703, *p* < 0.0001). The P1 and P2 strains were only impacted by concentrations >15.6 µg/mL (Figure 8A). Biofilm formation in the NCTC747 strain was influenced by a ZnO-NP concentration of 15 μg/mL (F (1.731, 10.39) = 16.12, *p* = 0.0009). The clinical strains of *P. acnes* were only affected by ZnO-NP titres >31.25 μg/mL (Figure 8B).

#### 3.3.4. Inhibitory Effect of Silver and Zinc Oxide Nanoparticles on Established *P. acne* Biofilm

**Figure 9:**
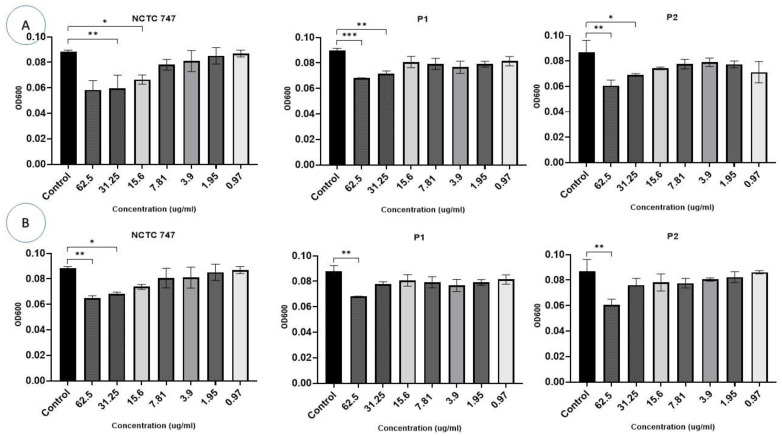
Impacts of different concentrations (0.97–62.5 µg/mL) (*X*-axis) of Ag-NPs (**A**) and ZnO-NPs (**B**) on biofilm eradication in the *P. acne* strains, NCTC747, P1, and P2, using CV staining after a 72 h incubation period and measuring OD at 600 nm (*Y*-axis). *** *p* < 0.0001, ** *p* < 0.001, and * *p* < 0.01.

In the experiments that evaluated the impact of Ag-NPs on two-day-old pre-formed biofilms, a larger concentration of Ag-NPs was necessary for the eradication of a CV-stained *P. acnes* biomass. CV staining was significantly reduced in all strains at a Ag-NP concentration >31.25 µg/mL. The metabolic activity of the cells in the pre-formed biofilms was largely influenced by Ag-NPs at concentrations ≥15.6 µg/mL (F (10, 66) = 29.27, *p* < 0.0001) (Figure 9A). Using Ag-NP doses of 15.625–62.5 µg/mL significantly decreased the metabolic activity of each strain (Figure 9A).

The eradication assays that were used to evaluate the treatment of pre-formed biofilms demonstrated an overall reduction in biofilm biomass in all strains exposed to ZnO-NPs compared to the controls (F (20, 66) = 9.148, *p* < 0.0001), although this effect was not consistent. Concentrations of 31.25 μg/mL and 62.5 μg/mL significantly decreased CV staining in the *P. acnes* biomass of the NCTC747 strain. Concentrations of 62.5 μg/mL also decreased CV staining in P1 and P2 strains (Figure 9B).

**Figure 10:**
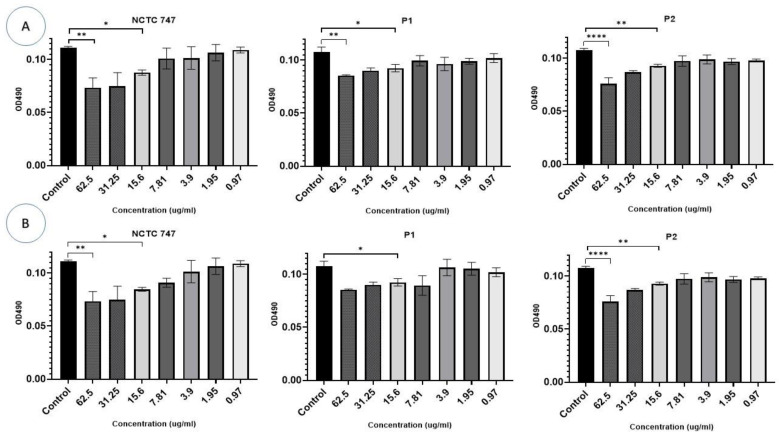
Impacts of different (A) Ag-NP and (B) ZnO-NP concentrations (0.97–62.5 µg/mL) (*X*-axis) on biofilm eradication of the *P. acne* strains, NCTC747, P1, and P2, using TCC staining after a 72 h incubation period and measuring OD at 490 nm (*Y*-axis). **** *p* < 0.0001, ** *p* < 0.001, and * *p* < 0.01.

The metabolism of the pre-formed biofilm community of *P. acne* strains incubated with Ag-NPs was inhibited (F (7, 8) = 6.741, *p* = 0.0076). Concentrations of ≥15.6 µg/mL of either Ag-NPs or ZnO-NPs were needed to inhibit the metabolic activity of all *P. acnes* strains (Figure 10A,B).

## 4. Discussion

*P. acnes* is one of the most abundant bacteria present on human skin. Although acne is not a contagious condition, an earlier study has highlighted the involvement of *P. acnes*, a Gram-positive bacterium, which resides on the pilosebaceous unit [54]. The continuous therapeutic application of antibiotics can cause them to become ineffective against *P. acnes* owing to the development of antibiotic resistance [10,11,12,55]. Employing NPs as a treatment for *P. acnes* may have clinical utility considering the increasing emergence of bacteria demonstrating resistance to traditional antibiotics. The aim of this study was to observe the anti-microbial properties of Ag-NPs and ZnO-NPs in relation to the growth of *P. acnes*. Three strains were investigated, i.e., two *P. acnes* clinical isolates, and the reference strain, NCTC747.

In this research, NPs were manufactured in conjunction with a seed extract obtained from *P. harmala.* The latter provided an economical source of both reducing and capping agents, which stabilise the NPs produced, facilitate the use of an environmentally friendly green synthesis approach, and restrict the toxic by-products and chemical waste substances created by chemical production methods [56,57].

In this study, the presence of both phenolic compounds and flavonoids was demonstrated in the extract produced from *P. harmala.* As secondary metabolites, phenolic compounds are characterised by their redox potentials which facilitate their ability to scavenge radicals and confer an antioxidant action which enables them to be utilised as reducing agents, hydrogen donors, and singlet oxygen quenchers, as well as for the chelation of metals [58]. Flavonoids, saponins, tannins, compounds reducers, volatile oils, anthraquinones, triterpenes, sterols, and alkaloids have been demonstrated in extract of *P. harmala* [59]. Therapeutic properties have been attributed to several of the chemical substances present [42,60]. The spectrum of phytochemical components found in plants reflects their practically unrestricted capacity to manufacture aromatic compounds and their derivatives. The latter have frequently been utilised by healers for their pain-relieving and antiseptic properties, as well as their activity against malaria and bacterial pathogens [61].

In the current study, green synthesised Ag-NPs and ZnO-NPs were produced using *P harmala* extracts, which function as both reducing and stabilising agents [62]. The formation of NPs was evidenced by the alteration in colour of the silver nitrate solution, transitioning from a brightly coloured orange to a deep reddish-brown colour for AgNPs, and from a light brown colour to a yellowish-black colour for ZnO-NPs. The results were consistent with earlier research [63,64].

The production of NPs was confirmed using UV–visible spectroscopy, which proved to be an effective way to confirm the synthesis of NPs [65]. The Ag-NPs and ZnO-NPs exhibited absorption peaks at around 460 and 399 nm, respectively; this is related to SPR in the case of AgNPs and due to the transition from valence band to conduction band in ZnO-NPs. The results obtained were consistent with the research conducted by Singh, Kim [66], and Pryshchepa and Pomastowski [67], which indicated that AgNPs and ZnO-NPs exhibited spectral bands within the range from 450 to 500 nm and from 350 to 450 nm, respectively. Upon comparing our findings with the prior results, it was discovered that both Ag-NPs and ZnO-NPs were successfully created.

The Ag-NPs exhibited a lower particle size compared to the ZnO-NPs, along with a more limited range of particle sizes. This observation was supported by the computed standard deviation (SD) obtained from the measurement of particle sizes. The DLS results validated the analysis of UV spectra for both AgNPs and ZnO-NPs.

The X-ray diffraction (XRD) peaks exhibited a strong correlation with the crystalline structure of Ag-NPs and ZnO-NPs. The examination revealed the degree of purity in the manufacturing of nanoparticles. The diffraction peak positions exhibited an identical pattern to that observed in the JCPDS.

The stability of NPs is governed by their zeta potential values [68]. These values indicate that the surface of the NPs has a negative charge, allowing them to disperse in the medium with a high level of stability. Particles with zeta potential values ranging from +35 to −35 mV are classified as a stable suspension, according to reports [69]. This suggests that our NPs were in a state of stability. Therefore, the presence of negative charges prevents the agglomeration of particles by creating an electric repulsion force between molecules with identical negative charges. This force stabilises the particles in their colloidal solution [70]. The SEM data validated the production of NPs and demonstrated their approximately spherical morphology and consistent dimensions.

The data revealed in this investigation show that the Ag-NPs and ZnO-NPs produced using this green synthesis technique could have significant anti-bacterial properties, as indicated specifically by the MIC and MBC values obtained via the broth microdilution method. It was observed that at low concentrations, both types of NPs were biocidal and effective against the anaerobic bacterium, *P. acnes*. Additionally, a disc diffusion anti-microbial activity assay demonstrated relatively high anti-bacterial activity in comparison to doxycycline, based on the size of the inhibition zones. Numerous investigations have documented the strong anti-bacterial activity of Ag-NPs and ZnO-NPs [71,72,73,74]. This arises because the small particle size creates a high surface/volume ratio, which allows NPs to permeate membranes more effectively and consequently to disturb bacterial metabolism and cell structure [75]. Despite the fact that both Ag-NPs and ZnO-NPs demonstrated powerful anti-microbial actions in the assays evaluating growth and MIC, in general, the effectiveness of the Ag-NPs was superior with respect to the three *P. acne* strains as a lower concentration proved to be efficacious.

Interestingly, this study’s findings also revealed that Ag-NPs and ZnO-NPs have a dose-dependent effect, which prevents the development of *P. acnes*-related biofilms in vitro. It has been demonstrated that established biofilms degrade when exposed to Ag-NPs and ZnO-NPs, despite the fact that many anti-microbials are less effective at killing bacteria enmeshed in biofilms [76]. *P. acnes* has also been shown to be more resistant to antibiotics when it is present as a biofilm rather than in the planktonic development phase [77,78].

A number of researchers have proposed that biofilm is predominantly disrupted as a result of Ag-NPs attaching to the EPS matrix. Recognition of the bacterial membrane peptidoglycan moieties leads to physical disruption, the liberation of ions, and the synthesis of reactive oxygen species (ROS), thereby generating oxidative stress and injuring chromosomal material [79]. The administration of Ag-NPs leads to alterations in the morphology in the structure of the biofilm, e.g., the creation of irregularities on the cell surface which infer the presence of cell lysis [80], cell wall disturbances, corrugation damage to the membrane, polarisation, and permeability variations within the membrane, and the generation of a specific EPS matrix which encompasses the strains of the organism [81]. Bacterial membrane breaches may also occur as a result of electrostatic bond formation with Ag-NPs, enabling them to pass through into the established biofilm [82].

The mechanisms responsible for the anti-bacterial actions of ZnO-NPs remain unclear. It has been postulated that NPs may injure the membrane of bacteria through perforation, penetration, or cellular uptake. Cytoplasmic contents may consequently escape and lead to cellular demise [83,84]. Following internalisation, the production of ROS may be promoted by ZnO-NPs, or Zn ions may be liberated which cause injury to genetic material, mitochondria, proteins, or other key cellular constituents essential for bacterial viability [85,86]. If NPs become associated with the membrane of bacteria, the NP–cell wall interface becomes a site for ROS manufacture. This process is promoted by irradiation with light and is the principal mode of bactericidal activity [87]. ROS produced and accrued precipitate oxidative stress-induced plasma membrane injury. The larger surface area of smaller NPs enables a higher concentration of ROS to be generated in proximity to the cell wall and consequently enhances the bactericidal effect [19].

Our findings show that the metabolic activity of the strains of *P. acnes* was diminished following the addition of both ZnO-NPs and Ag-NPs; the liberation of Ag and Zn ions could underlie this observation [88,89]. The inhibition of such cellular activities would inevitably lead to impaired bacterial cell function. Consequently, the free metal ions cause the demise of the bacterial cell via toxicity [90,91].

The current results demonstrate a higher efficacy of the Ag-NPs, in comparison to ZnO-NPs, in suppressing biofilm development or destroying biofilm. The Ag-NPs had larger growth inhibition diameters but were of a smaller dimension than the ZnO-NPs. Typically, the anti-bacterial or biomedical characteristics of NPs reflect the production technique and conditions, e.g., the reducing agent used and temperature, as well as the strength applied and their dimensions [33,34]. The identical reducing agent for each NP type was utilised for this study, and so the anti-biofilm and anti-bacterial properties observed may be partially a result of NP dimension disparity [92]. For the NPs that are of a greater dimension, they form clusters at the outer membrane of bacterial pathogens, and ROS manufacture forms the principal anti-microbial mechanism, the latter being able to injure the bacterial membrane [93]. The greater surface area of smaller NPs facilitates a higher degree of ROS production and, consequently, a more potent anti-bacterial effect [94]. Earlier studies have shown that NPs of smaller dimensions congregate in proximity to pathogens [92]. Consequently, their anti-microbial effect is enhanced as ROS are generated adjacent to the bacterial membrane. The ability of NPs to breach cell membranes and to enter the cell also facilitates the catastrophic intracellular manufacture of ROS, thereby promoting smaller NPs to a state of higher bactericidal efficacy [92].

Ions are liberated from the surface of intracellular NPs and become bound to key cell constituents and nutrients, resulting in a cytotoxic effect [95]. Although the true mechanism through which NPs negatively impact bacteria merits additional study, the current findings suggest that the dimension of the NPs is a factor governing their bactericidal activity. ZnO-NPs, with a spherical morphology and diameter of 6 nm, were demonstrated to have greater efficacy than 55 nm long ZnO nanorods when tested against *E. Coli* and *S. aureus* according [96].

Despite the fact that Ag-NPs and ZnO-NPs are promising anti-bacterial tools, concern remains regarding their adverse event profile in humans. The likelihood of cytotoxicity following their use has retarded their development for inclusion in chemotherapy regimens [97]. One study utilised the neutral red uptake test to determine the toxic profile of a spectrum of Ag-NP titres in HeLa cell lines [98]. At strengths of ≤120 µg/mL, no toxicity was demonstrated, but at 240 µg/mL, cytotoxicity was seen. This is a higher titre of Ag-NPs and ZnO-NPs than that investigated in the current study. Experiments performed on in vitro human cell cultures have identified toxic effects for Ag-NPs within the dose spectrum 10–100 µg/mL [99,100]. In the current study, bacterial inhibition was observed at lower does than those reported as toxic, which implies that there is the potential for the safe use of low concentrations of NPs.

To the best of the authors’ knowledge, large research studies have not yet been performed which have investigated the possible utility of NPs in the treatment of infection with *P. acnes.* A limitation of the current work was that only two strains of *P. acnes* were studied, and so only preliminary data could be presented. Additional studies are therefore required, for instance, to assess the impact of using NPs either admixed with or containing conventional anti-bacterial agents. The current study presents some basic data which substantiate the use of NPs against biofilms of *P. acnes.* This is highly pertinent to the ongoing difficulties created by the emergence of resistance to traditional anti-microbial agents and their control worldwide.

To date, unlike the issue with conventional antibiotics, there has been no emergence of bacterial resistance to NPs despite extended periods of exposure. The biocidal effects of NPs appear to focus on a number of loci within bacterial cells and therefore offer a broad activity spectrum. This may underlie the failure of bacteria to show resistance despite prolonged applications. The technique used for preparing Ag-NPs and ZnO-NPs was simple, eco-friendly, and cost-effective, and thus, the method is suitable for commercial upscaling. However, further studies are required to determine the safety of these green synthesised NPs in order to enable these agents to become established as a treatment for use in vivo. Further research is recommended.

It can be concluded that dilute titres of Ag-NPs and ZnO-NPs could have anti-bacterial effects and also exhibit anti-biofilm activity in the context of either de novo created or well-established biofilms. They therefore could have the potential to be an efficacious constituent in dermo-cosmetic therapies for acne. These results substantiate the future potential for Ag-NPs and ZnO-NPs to become established either as an anti-bacterial medication per se or as an adjunct to established anti-microbial therapies. It is suggested that future studies could evaluate the effects of NPs delivered alongside conventional anti-microbial agents. Such work could facilitate the implementation of NPs as an additional mode of treatment against infectious disease. In order to achieve this, additional investigations conducted both in vitro and on a molecular level are required to gain a greater understanding of the toxic effects of the released Ag and Zn metal ions. Additionally, longitudinal studies are merited to obtain a complete adverse event profile for these agents in the long term and to establish their potential for mutagenic or carcinogenic effects. This research is paramount in order to clarify that NPs could be implemented in the clinical domain without hazard. The way in which both Ag-NPs and ZnO-NPs interact with innate system immune cells could also be assessed by future research, as this may influence their anti-microbial potency and effect on established biofilms.

## Figures and Tables

**Table 1:** Impact of Ag-NPs and ZnO-NPs on *P. acnes* strains as demonstrated by the well diffusion assay. The average ± standard deviation scores from three experiments were used to determine each value in the table. Positive control, 30 µg doxycycline; negative control, distilled water.

	Zone of Inhibition (mm)			
	Ag-NPs (250 µg/mL)	ZnO-NPs (250 µg/mL)	Positive Control	Negative Control
*P. acnes* NCTC747	13.2 (0.8)	11(1.0)	16.7 (0.3)	0.0
P1	11.7 (0.8)	9.7(1.2)	13.0 (0.5)	0.0
P2	11.0 (0.5)	9.7(0.6)	14.0 (0.5)	0.0

## Data Availability

The datasets used and/or analysed during the current study are available from the corresponding author on reasonable request.

## Supplementary Materials

The following supporting information can be downloaded at: https://www.mdpi.com/article/10.3390/ph17020255/s1, Figure S1: color change in the reaction mixture indicating formation of Ag-NPs and ZnO-NPs; Table S1: Biofilm forming ability of *P.acne*
