# Supplementary material for: Anti-Bacterial Activity of Green Synthesised Silver and Zinc Oxide Nanoparticles against Propionibacterium acnes
Source: Pharmaceuticals (Basel). 2024 Feb 16;17(2):255. doi: 10.3390/ph17020255 (PMC10891609; doi:10.3390/ph17020255)
Supplement: Supplementary file 1 [file pharmaceuticals-17-00255-s001.zip › pharmaceuticals-2826794-supplementary.pdf]

Anti-bacterial activity of green synthesised silver and zinc oxide nanoparticles against *Propionibacterium acnes*

Hafez Al-Momani[1]*, Muhannad I. Massadeh[2], Muna Almasri[2], Dua'a Al Balawi[3] , Iman Aolymat[4] Saja Hamed[5], Borhan Aldeen Albiss[6], Lugain Ibrahim[3], Hadeel Al Balawi[3], Sameer Al Haj Mahmoud[7].

Supplementary figure 1: color change in the reaction mixture indicating formation of Ag-NPs and ZnO-NPs

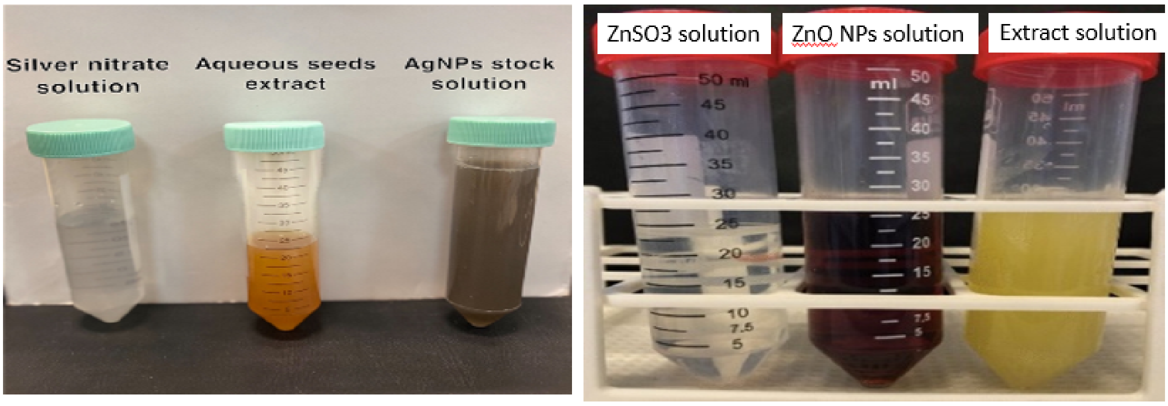

	Odc	OD of positive control	2*Odc	4*Odc	
ATCC	0.096	0.294	0.192	0.384	moderate-biofilm producer
PA1	0.058	0.381	0.116	0.232	strong-biofilm producer
PA2	0.062	0.34	0.124	0.248	strong-biofilm producer

Supplementary table 1: Biofilm forming ability of *P.acne*
